# Host induced gene silencing of *Magnaporthe oryzae* by targeting pathogenicity and development genes to control rice blast disease

**DOI:** 10.3389/fpls.2022.959641

**Published:** 2022-08-11

**Authors:** Mengying Wang, Ralph A. Dean

**Affiliations:** Fungal Genomics Laboratory, Department of Entomology and Plant Pathology, Center for Integrated Fungal Research, North Carolina State University, Raleigh, NC, United States

**Keywords:** rice blast, host-induced gene silencing, transgenic plant, RNA silencing, cross-kingdom communication

## Abstract

Rice blast disease caused by the hemi-biotrophic fungus *Magnaporthe oryzae* is the most destructive disease of rice world-wide. Traditional disease resistance strategies for the control of rice blast disease have not proved durable. HIGS (host induced gene silencing) is being developed as an alternative strategy. Six genes (*CRZ1, PMC1, MAGB, LHS1, CYP51A, CYP51B*) that play important roles in pathogenicity and development of *M. oryzae* were chosen for HIGS. HIGS vectors were transformed into rice calli through *Agrobacterium*-mediated transformation and T0, T1 and T2 generations of transgenic rice plants were generated. Except for *PMC1 and LHS1*, HIGS transgenic rice plants challenged with *M. oryzae* showed significantly reduced disease compared with non-silenced control plants. Following infection with *M. oryzae* of HIGS transgenic plants, expression levels of target genes were reduced as demonstrated by Quantitative RT-PCR. In addition, treating *M. oryzae* with small RNA derived from the target genes inhibited fungal growth. These findings suggest RNA silencing signals can be transferred from host to an invasive fungus and that HIGS has potential to generate resistant rice against *M. oryzae*.

## Introduction

With finite agricultural resources and an increasing human population (Ramankutty et al., 2018), new approaches are in high demand to support sustainable food production. Rice (*Oryza sativa* L.), which is the staple food for over half of the world’s population and provides more than 1/5 of the calories consumed by humans, is crucial for global food security (Seck et al., 2012). Worldwide production of milled rice was forecasted at a record of 510.7 million tons in 2021/22 (Childs and LeBeau, 2021). However, diseases severely threaten rice production. Rice blast disease, caused by the hemi-biotrophic fungus *Magnaporthe oryzae* (teleomorph of *Pyricularia oryzae*), leads to annual yield losses worth billions of dollars, enough to feed 60 million people (Dean et al., 2005; Talbot and Wilson, 2009). In addition to rice, the fungus also infects approximately fifty grass and sedge species (Ou, 1985). Eighty-five countries throughout the world have reported the presence of this disease, and it is likely to present practically everywhere that rice is grown commercially (Ou, 1985). More effective control of blast disease would constitute a major contribution to ensuring global food security.

Rice blast disease is initiated by a three-celled tear shaped conidium which germinates once attached to the plant surface. A melanized appressorium at the tip of the germ tube forms a penetration peg, which pushes through the plant cuticle and cell wall. Primary hyphae differentiate as bulbous hyphae in the epidermal cells before emerging as invasive hyphae, spreading and causing lesions on aerial tissues. Within 5–7 days lesions discharge numerous conidia enabling new infections (Hamer et al., 1988; Dean et al., 2005; Talbot and Wilson, 2009). The pathogen can infect and cause lesions on all above-ground plant tissues such as leaves, nodes, culms, panicles, peduncles and grains. Infection can occur at the seedling stage, the entire vegetative phase as well as the reproductive phase. To manage this disease, crop rotation, proper fertilization in addition to irrigation are highly recommended. Chemical fungicides are also used, but none are considered to be highly successful. Genetic resistance is the most effective way for rice blast disease control, however, resistance strategies employed to date are generally not durable (Ou, 1980; Zeigler et al., 1994; Dean et al., 2012).

Plants, unlike animals, do not possess circulating immune cells, but are equipped with two interconnected tiers of receptors for the recognition of pathogens and subsequent defense responses. The first tier governed by pattern recognition receptors (PRRs) is triggered by pathogen-associated molecular patterns (PAMPs) and is known as PAMP-triggered immunity (PTI). Successful pathogens further secrete effectors to subvert PTI and facilitate infection. In response, host plants have evolved resistance (R) genes that recognize pathogen effectors and induce the second tier of defense: effector-triggered immunity (ETI) (Jones and Dangl, 2006; Dangl et al., 2013). Breeding rice varieties carrying R genes are used for blast disease control. Over 100 major blast R genes have been identified and more than 30 of them have been cloned (Wang et al., 2017). Major blast R genes such as *Pita*, *Ptr, Pi-z*, *Pi-b* and *Pikh, Pikm, Piks* have been deployed in US rice varieties since 1960 (Jia et al., 2019). Although incorporation of R genes into rice has proved to be an effective and environmentally friendly strategy for blast resistance breeding (Singh et al., 2012; Wang and Valent, 2017), many years are required to develop new cultivars. Moreover, the pathogen is capable of rapid genetic changes through mechanisms such as active transposable elements, random mutation and recombination to “break-down” R gene resistance within 2 or 3 years after planting (Wang and Valent, 2017). Furthermore, many R genes may also be associated with yield loss or nutrition defects and consequently may not be commercially popular (Brown, 2002; Burdon and Thrall, 2003). Therefore, innovative strategies are needed for durable and effective resistance. The concept of parasite-derived resistance (PDR) which confers host resistance by expressing parasite genes in the host plant was proposed decades ago as a radical approach but only since the discovery of small RNA silencing mechanism is this now a possibility (Sanford and Johnston, 1985).

With several notable exceptions such as budding yeast, RNA interference (RNAi) and its role in chromatin regulation are largely conserved throughout all eukaryotes (Shabalina and Koonin, 2008; Drinnenberg et al., 2009). Small non-coding RNA can induce silencing of target genes at the transcriptional and post-transcriptional levels (Ghildiyal and Zamore, 2009). It is usually initiated by processing double stranded RNA (dsRNA) into short RNA duplexes through the action of “Dicer” (RNase III), followed by the cleavage, and degradation or translational repression of targeted mRNA through the RNA-induced Silencing Complex (RISC) (Ghildiyal and Zamore, 2009). RNAi mechanisms have been widely exploited and used as a powerful genetic tool to silence genes of interest in target organisms (Tang and Galili, 2004; Tan and Yin, 2005; Saksmerprome et al., 2009; Das and Sherif, 2020).

Conceptually, host-induced gene silencing (HIGS) is performed by expressing an RNAi construct in a host plant resulting in production of an RNA silencing signal that will be absorbed by the invading pathogen and silence the target pathogen gene. This concept was successful for control of root-knot nematodes and of lepidopteran and coleopteran insects feeding on transgenic plants carrying RNAi constructs targeting corresponding pest genes (Huang et al., 2006; Baum et al., 2007; Mao et al., 2007). HIGS has been demonstrated in several different fungal patho-systems. HIGS was first reported by Nowara and coworkers in 2010, where HIGS in barley (*Hordeum vulgare*) was evaluated against the obligate pathogen powdery mildew fungus *Blumeria graminis*. Host-induced gene silencing of the effector gene *Avra10* resulted in reduced fungal development in the host plant (Nowara et al., 2010). In addition to being effective against obligate biotrophic and hemi-biotrophic fungi and Oomycetes, where intimate contact is established with living plant cells, HIGS is also reported to be effective against necrotrophic fungal pathogens such as *Fusarium oxysporum* and *Rhizoctonia solani* (Govindarajulu et al., 2015; Hu et al., 2015; Sanju et al., 2015; Zhou et al., 2016). HIGS has also been shown to reduce the synthesis of toxic secondary metabolites, demonstrating its potential for precise engineering for food security (Thakare et al., 2017). Resistance conferred by HIGS may be more durable due to the vital function of genes that can be targeted such as genes encoding chitin or cellulose synthase, cytochrome P450 lanosterol C14a-demethylase in fungi (Koch et al., 2013; Cheng et al., 2015; Jahan et al., 2015; Andrade et al., 2016).

Though HIGS has been shown to be successful in a number of patho-systems (Nowara et al., 2010; Panwar et al., 2013; Hu et al., 2015; Zhou et al., 2016; Qi et al., 2018; Song and Thomma, 2018; Xu et al., 2018; Dou et al., 2020; Raruang et al., 2020), its applicability for control of rice blast disease has not been extensively explored (Guo et al., 2019; Li et al., 2020). This study was conducted to assess the effectiveness of targeting 6 key genes regulating growth and pathogenicity using HIGS for rice blast disease control. To accelerate these studies, we employed a vector that enabled visual detection of transgenic material. As small silencing RNAs have been illustrated to transfer bi-directionally between plants and fungi (Weiberg et al., 2013; Wang et al., 2016; Cai et al., 2018), the mechanism(s) of how they achieve cross-kingdom transportation is still unclear (Wang and Dean, 2020). In this study, we also investigated the hypothesis that RNA silencing signals may be absorbed by *M. oryzae*.

## Materials and methods

### Vector construction

Six genes of *M. oryzae* were selected as targets for HIGS (Table 1). Gene annotation and sequence information were obtained from the database ‘‘EnsemblFungi’’^1^. Invitrogen online RNAi designer tools were used to choose the optimal silencing fragment for target gene (Invitrogen^2^). Selected fragments were checked using the BLAST tool of the rice database ‘‘Rice genome annotation project’’ to confirm the absence of identical sequence (maximum length of identity less than 19nt) in the rice genome to ensure target fragments were specific to *M. oryzae*^3^. Threshold of expected value (E-value) is 10.

**TABLE 1:** Candidate host-induced gene silencing (HIGS) genes of *Magnaporthe oryzae.*

Gene name	Gene ID	Target fragment “Start-Stop (bp)”	Fragment length (bp)	Description of encoded protein
*CRZ1*	MGG_05133	1355–1824	470	Calcineurin responsive transcription factor
*PMC1*	MGG_02487	135–915	781	Calcium-transporting ATPase 2 (Vacuolar Ca^2+^-ATPase)
*MAGB*	MGG_00365	65–697	633	Guanine nucleotide-binding protein subunit alpha
*LHS1*	MGG_06648	858–1325	467	ER chaperon, essential for translocation of secreted protein across ER membrane
*CYP51A*	MGG_04628	493–794	302	Cytochrome P450 51, essential for ergosterol biosynthesis, membrane integrity
*CYP51B*	MGG_04432	268–452	206	Cytochrome P450 51, essential for ergosterol biosynthesis, membrane integrity

Primers were designed to generate PCR amplicons around 200- to 500- base pair (bp) length corresponding to exons of selected genes. The primer sequences used for the preparation of HIGS constructs are listed in Supplementary table 1. Flanking sequence and sequence of attB recombination sites were added to 5’end of each primer.

*Magnaporthe oryzae* strain 70-15 was cultured in liquid complete media (1L contains 10 g sucrose, 6 g casamino acid, 6 g yeast extract and 1 ml *Aspergillus nidulans* trace elements liquid) for 3 days and then mycelia were collected for RNA extraction. RNA was isolated using the RNeasy plant mini kit (Qiagen, Hilden, Germany) following the manual instruction. Total RNA was then pretreated with RNase-free DNase I (NEB, Ipswich, MA, United States) and subjected to reverse transcription using the Reverse Transcription System (Promega, Madison, WI, United States) based on manual instruction.

PCR reaction was performed in a total volume of 25 μL using standard *Taq* polymerase from NEB company to obtain target gene fragments. PCR parameters (primer and template annealing time, annealing temperature, final extension time) were optimized for each candidate gene. PCR mix with total volume of 25 μL contained 50 ng of *M. oryzae* cDNA, 0.4 μM of forward primer and 0.4 μM of reverse primer, 200 μM of dNTPs, 2.5 μL of 10X buffer, Nuclease free water and 0.625 units of *Taq* DNA polymerase. The PCR amplification conditions were as follows: 95°C for 5 min; 35 cycles of 95°C for 30 seconds, 55°C for 30 seconds and 72°C for 45 seconds; 72°C for 10 min and hold at 4°C (annealing temperature ranged from 53–58°C). For genes combination *CYP51A* and *CYP51B*, two amplicons were linked into one by double joint PCR. PCR products were subsequently confirmed by size-fractionation on 1.5% agarose gels and were purified by kit, eluted with 30 μl of water (QIAquick Gel Extraction Kit, QIAGEN).

Vector pDONOR221 was used as ENTRY vector for the “Gateway cloning system”. After purification of the PCR products, target fragments of each *M. oryzae* gene were inserted to the entry vector pDONR221 (Invitrogen) through BP recombination cloning individually. Then the pDONR221 carrying two recombination sites for “LR Clonase reaction” was transformed to *Escherichia coli* DB3.1 competent cell. Plasmids purified by a minipreparation kit (QIAprep Spin Miniprep Kit, QIAGEN) were further confirmed by PCR amplifying corresponding target regions.

For HIGS expression, pBDL03 (gift from Dr. Shaohong Qu) was used as the destination vector (Zhao et al., 2021). This vector is derived from the pANDA vector (Okano et al., 2008) and designed so that each target fragment is inserted into two regions flanked by recombination sites in opposite directions, resulting in inverted repeat sequences linked by *Gus* gene (Fragments were separated by *Gus* linker, *Gus* linker sequence can be found on website^4^), controlled by maize (*Zea mays*) ubiquitin promoter and T3A terminator (Miki and Shimamoto, 2004; Okano et al., 2008). The vector contains the hygromycin resistance gene under control of “Cauliflower Mosaic Virus (CaMV) 35S promoter” and 35S terminator for selection in rice (Zhao et al., 2021). In addition, the vector contains *mCherry* gene (Subach et al., 2009) to facilitate visual confirmation of transgenic plants (Zhao et al., 2021). This gene was under control of a CaMV 35S promoter and ‘‘*Agrobacterium* Nopaline Synthase terminator’’ (tNOS). The target sequences were inserted in the destination vector through ‘‘LR recombination’’ using ‘‘Gateway LR clonase II enzyme Mix’’ (Invitrogen/life technologies). Manufacture’s guide was followed while the incubation time for recombination was extended to overnight. Plasmids obtained from the LR reaction were transformed to *E. coli* DB3.1 competent cells. Positive plasmids purified by minipreparation (QIAprep Spin Miniprep Kit, QIAGEN) were confirmed by amplifying corresponding target fragments as well as size fraction check on agarose gel after restriction enzyme (*Kpn*I and *Sac*I from NEB company) digestion. DNA sequencing was applied for final confirmation and MEGA4^5^ software was used for sequence processing and alignment.

*Agrobacterium tumefaciens* EHA101 was cultured in liquid and solid YEP media (1L contains 10 g yeast extract, 10 g peptone, 5 g NaCl, 15 g agar for solid media). Positive plasmids were transformed into agrobacteria by electroporation.

### Rice transformation

Rice (*Oryzae sativa* ssp *japonica* cv Nipponbare) was used in this study. Rice callus was induced from the embryos of mature seeds and transformation was conducted using the *Agrobacterium*-mediated transformation method (Patel et al., 2013).

### Callus induction

Husk from rice seeds were removed, and seeds were then sterilized by using 75% ethanol for 1min, then 50% bleach (3% sodium hypochlorite) for 30 min, followed by washing 3 times with sterile distilled water. Seeds without husks were cultured on callus induction media (4g/L N6 salts, 1 mg/L Thiamine-HCL, 250 mg/L Myo-inositol, 1 g/L Casein hydrolyzate, 690 mg/L Proline, 30 g/L Maltose, 5 mg/L 2,4-D, 1 mg/L BA, pH 5.9). Friable calli were transferred to fresh callus induction medium every 2–3 weeks.

### Transgenic rice generation

The following media were used. Infection media: 2.2 g/L MS salts, 1 mg/L Thiamine-HCL, 250 mg/L Myo-inositol, 1 g/L Casein hydrolyzate, 690 mg/L Proline, 30 g/L Glucose, 1 mg/L 2,4-Dichlorophenoxyacetic acid (2,4-D), 200 uM acetosyringone, pH 5.4; Co-culture media: same as callus induction media with 60 mg/L maltose and 200 μM of acetosyringone; Selection media: same as callus induction medium with 200 mg/L Timentin and appropriate selective agent (Hygromycin 50 mg/L) and reduced 2,4-D (2 mg/L); Regeneration media: 2.2 g/L MS salts, 1 mg/L benzyl adenine (BA), 30 g/L Maltose, 3 g/L Phytagel with 200 mg/L Timentin and appropriate selective agent (reduced concentration, Hygromycin 20 mg/L); Rooting media: 2.2 g/L MS salts, 30 g/L Maltose, 3 g/L Phytagel with 200 mg/L Timentin and appropriate selective agent (Hygromycin 50 mg/L).

*Agrobacteria* carrying the destination vector were cultured in 5 mL YEP medium at 28°C overnight followed by transfer to 45 mL MS infection media. *Agrobacteria* were grown at 28°C for another 2–3 h until the optimal OD value was achieved (OD600: 0.5–0.6). Then 2 to 4 months old calli were mixed with the *Agrobacteria* culture for 3 min at 42°C followed by 10 min incubation at room temperature. After incubation with *Agrobacteria*, callus was blotted on three layers of sterile filter papers to get rid of excessive *Agrobacteria* suspension and then cultured onto co-culture medium, incubated at 25°C in a growth chamber without light for three days. Callus were further transferred to selection medium and incubated in a growth chamber at 25°C in dark for 2 weeks. Callus on hygromycin-based selection medium were sub-cultured every 3 weeks. Calli were visually inspected as well as by epi-fluorescence microscopy (SMZ1000, Nikon, Melville, NY, United States) for mCherry signal. After 2–3 times of sub-culture, calli were transferred to shoot and root regeneration media. Rooted plantlets were transplanted to soil and grown in the greenhouse.

### Plant materials and growth conditions

Transgenic rice plants as well as non-transgenic control plants (*Nipponbare* wild type) were grown in a Greenhouse at North Carolina State University with a temperature range from 20°C (night) to 38°C (day). Plants were also maintained in a growth chamber in the NCSU Phytotron at 26°C with a 12-h light/dark photoperiod. The transgenic plants (T0) were self-pollinated and T1 seeds were collected. T1 plants were grown to obtain T2 progeny.

For rice growth, seeds of the T0, T1, and T2 transgenic plants as well as wild type plants were always de-husked first and sterilized by immersion in 75% ethanol for 1 min followed by 3% sodium hypochlorite for 30 min. After three times washing with sterile water, seeds were germinated on half-strength Murashige and Skoog media and incubated for around 10 days before transferring to pots.

### Genomic DNA isolation and genotyping confirmation of transgenic rice

Transgenic plants were initially evaluated under fluorescence microscope (SMZ1000, Nikon, Melville, NY, United States) to screen for positive transgenic rice. Transformation was confirmed in all transgenic lines using gDNA PCR to amplify the target fragments presenting with opposite direction on the left and right sides of *Gus* linker gene on the destination vector. Primer pairs “Gus3 & corresponding target gene primer” were used to amplify fragment inserted on the left border of *Gus* gene while primer pairs “Gus4 & corresponding target gene primer” were used to amplify fragment inserted on the right border of *Gus* gene.

Genomic DNA was extracted from young leaves of transgenic rice using the CTAB method. PCR analysis was performed in a final volume of 25 μL containing 1 μL of forward primer (Gus3 primer or Gus4 primer) (10 μM), 1 μL of reverse primer (target gene reverse primer) (10 μM), 1 μL of dNTPs (10 μM), 2.5 μL of 10 × buffer and 0.125 μL of *Taq* DNA polymerase from NEB company. Fifty ng of genomic DNA was used as template. The PCR amplification conditions were as follows: 95°C for 5 min; 30 cycles of 95°C for 30 seconds, 55°C for 30 seconds and 72°C for 45 seconds; 72°C for 10 min and hold at 4°C.

### Pathogenicity assay and disease assessment

Rice seeds were de-husked and surface sterilized before cultured on half MS media for germination. Seedlings were screened using fluorescence microscope (SMZ1000, Nikon, Melville, NY, United States) for positive transgenic plants based on the *mCherry* marker gene carried by the destination vector. Seedlings with roots and shoots showing red fluorescence under fluorescence microscope were selected as positive transgenic plants. Genomic DNA PCR was further conducted to confirm positive transgenic plants and non-transgenic segregants. Positive transgenic plants were used for subsequent “pathogenicity assay” while non-transgenic segregants were used as negative control.

Rice seedlings were then transferred to pots and incubated at 26°C with a 12-h light/dark photoperiod. All inoculations and disease evaluation were conducted in the Phytotron facilities at North Carolina State University. Pathogenicity assay was conducted for both the T1 generation and T2 generation rice plants. At least 15 plants per transgenic line were used each time and at least 5 leaves were inoculated for each rice plant.

Strain *M. oryzae* KJ-201 was used for inoculation. *M. oryzae* was grown on oatmeal agar media (Difco, Detroit, MI, United States) for 2–3 weeks at 24°C in the dark. Conidial production was induced by scratching the plate surface with a sterilized loop and placing the plate after scratching under continuous fluorescence light for 1 week for sporulation.

Two-month-old rice plants were inoculated with *M. oryzae* spore suspension at the concentration of 5 × 10^5^spores/mL containing 0.05% Tween-20 (Park et al., 2012). Fully expanded rice leaves were selected for inoculation. A 2- to 3-inch-long piece of transparent Scotch tape (3M company, MN, United States) was placed perpendicular on the rice leaf. Leaves were wounded using a mouse ear punch (Kent Scientific Corporation, CT, United States) in the middle of the leaf at the site of tape attachment. Then, a 10 μL of spore suspension was added to the press-injured site. Both sides of the inoculated site were wrapped with tape to hold the spore suspension and maintain humidity. Inoculated plants were placed in black plastic bags in a growth chamber to block light and retain humidity at 25°C. Bags were removed after 24 h. Plants were then incubated in the growth chamber under 12 h light, 12 h dark at 25°C.

Disease lesions were recorded around 2 weeks after inoculation and photographed. Necrotic tissue size was measured by analyzing the photographs with ‘‘ImageJ’’ software^6^ (Park et al., 2012). Data was analyzed statistically using ‘‘Graphpad-Prism’’ software^7^.

### RNA isolation, cDNA synthesis and quantitative real-time PCR (qRT-PCR)

To examine fungal target gene’s transcription following infection of transgenic rice plants, leaves from transgenic rice inoculated with *M. oryzae* strain KJ-201 were collected for quantitative RT-PCR analysis. Two-month-old T1 and T2 generation plants were inoculated with *M. oryzae*, and total RNA was extracted from infected leaves 2 weeks after inoculation. Three to four leaves from the same infected plant were ground to a fine powder with liquid nitrogen using a precooled sterile mortar and pestle. Total RNA was extracted using the RNeasy plant mini kit (QIAGEN) or using TRIzol reagent (Invitrogen) according to the manufacturer’s instruction. RNA was pretreated with RNase-free DNase I (Invitrogen) to remove genomic DNA contamination, then first-strand of cDNA was synthesized using the reverse transcription system (Promega).

The cDNA was then diluted 20 times with distilled water and used as template for qRT-PCR, which was performed in a 96-well plate using the iQ5 real-time PCR detection system (Bio-Rad). Platinum SYBR Green qPCR SuperMix-UDG (Invitrogen) reagent was used. The reaction included 5 μL of SYBRGreen qPCR SuperMix, 1 μL of cDNA template from each sample, 0.5 μL of 10 μM primer, and 3 μL of distilled water. The thermal cycle was split into three stages: initiated by one cycle of 95°C for 20 seconds (hold stage), followed by 40 cycles of 95°C for 15 seconds, 60°C for 30 seconds (PCR stage), with a final melt curve stage: 95°C for 15 seconds, 60°C for 1 min and 95°C for 15 seconds.

*Magnaporthe oryzae* β-*Tubulin* gene and *M. oryzae ILV5* gene were tested as internal controls (Fudal et al., 2007; Wilson et al., 2010). Primers for target genes were also redesigned (sequence information in Supplementary Table 1). Three independent amplifications were performed for each primer set as technical replication.

Relative fungal target gene transcription was calculated using the 2^–ΔΔ*CT*^ method. ΔΔC_*T*_ represents (C_T, target_ - C_T, reference_) _transgenic rice_ - (C_T, target_ -C_T, reference_) _nipponbare_. Relative gene transcription was then calculated as a ratio represented by the equation 2^–ΔΔ*CT*^ (Livak and Schmittgen, 2001; Park et al., 2012). Statistical analysis was conducted using “GraphPad-Prism” software.

### Synthesis of artificial dsRNA

Entry vectors were used as templates to obtain PCR products for target genes. PCR products of control gene *EGFP* were obtained by using cDNA of *M. oryzae* strain KJ201-EGFP as template. The T7 promoter sequence was added to genes’ forward and reverse primers (Supplementary Table 1). The PCR products were further used as templates for corresponding dsRNA synthesis. Synthesis of dsRNA *in vitro* used the high-scribe T7 *in vitro* transcription system (Fermentas, Waltham, MA, United States). Template DNA was removed after *in vitro* transcription by using DNase I. RNA transcripts were purified and annealed in TE buffer at 65°C followed by snap cooling. The dsRNA concentration was determined by Nanodrop spectrophotometry. Synthesized dsRNA was stored at −80°C.

### Preparation of diced-siRNA pool

The dsRNA was digested with Short Cut RNase III (New England Biolabs, United States) to prepare diced-siRNA pools according to the manufacturer’s instruction. Briefly, 10 μg dsRNA were digested with Short Cut RNase III in a 100 μL reaction system (dH_2_O, 10X ShortCut Reaction Buffer: 10 μL, dsRNA: 10 μg, ShortCut RNase III: 10 μL, 10X MnCl_2_: 10 μL) at 37°C for 20 min. Then 10 μL 10X EDTA was added to stop the reaction. The diced small RNAs were purified by precipitation using nuclease-free glycogen, one-tenth volume of 3M sodium acetate (pH 5.2) and 3 volumes of cold ethanol. Diced siRNA were stored at -80°C (Kalleda et al., 2013).

### Treatment of siRNA from the target gene inhibited growth of *Magnaporthe oryzae*

*Magnaporthe oryzae* strain KJ-201 was cultured on oatmeal media and sporulation was induced using the method described above. Spores were washed from plates and filtered using two layers of cheesecloth. Concentration of spore suspension was adjusted to 200 spores/μL using distilled water and incubated for 12 h at room temperature. Synthetic siRNA was then added to diluted spore suspension to a final concentration of 30 nM of siRNA with 4 spores/μl. Suspension was incubated for another 24 h at room temperature and 100 μL of suspension was then inoculated onto solid complete media. Plates were incubated at 25°C for 5 days without light and then photographed. The diameter of fungal colonies was measured. For each synthetic siRNA treatment, a minimum of three replicates were included and the experiments were repeated three times.

## Results

### Selection of target fungal genes and design of experimental workflow

To investigate the potential of HIGS against *M. oryzae* in rice, six genes with different biological functions in *M. oryzae* were selected as candidate targets (Table 1). Calcium as an intracellular signaling molecule plays important roles in cellular development and pathogenicity in fungi. Silencing calcineurin encoding genes *PsCNA1/PsCNB1* in *Puccinia striiformis* f. sp. *tritici* through HIGS has been demonstrated to lead to slower extension of fungal hyphae and reduced production of urediospores (Zhang et al., 2012). In *M. oryzae*, the transcription factor *CRZ1* acts as a downstream regulator in calcium-dependent signaling pathway, and knockout mutants result in hypersensitivity to Ca^2+^, reduced numbers of conidia, abnormal appressoria and attenuated pathogenicity (Choi et al., 2009; Zhang et al., 2009). Systematic RNA silencing has also been conducted in *M. oryzae*, and 37 calcium-signaling related genes were targeted (Kadotani et al., 2003). Among those target genes, silencing of *PMC1* which is involved in a vacuolar membrane located Ca^2+^ pump can lead to reduced vegetative growth as well as sporulation (Kadotani et al., 2003). Thus, *CRZ1* and *PMC1* were selected for HIGS assay in this study.

Regulatory guanine nucleotide-binding proteins (G proteins) are involved in the signal transduction cascades which are important for proper regulation of cell function, division and differentiation (Liu and Dean, 1997). The G protein α subunit gene *MAGB* of *M. oryzae* is involved in signal transduction pathways controlling vegetative growth, conidiation, conidium attachment, appressorium formation, mating as well as pathogenicity (Fang and Dean, 2000). This gene was also selected as a promising candidate for HIGS.

Host colonization requires secretion of effector proteins into plant cells to suppress host immunity and promote pathogen growth. However, individual effector genes are not good candidates for silencing because pathogens usually secrete multiple effectors with overlapping function. Silencing conserved fungal genes that have essential biological functions, such as genes essential for effector secretion are more likely to confer host durable resistance. In *M. oryzae*, there are two distinct effector secretion pathways. First is the conventional Endoplasmic Reticulum (ER)-Golgi secretion pathway secreting apoplastic effectors (effectors that do not enter host cells, and generally remain within the extra invasive hyphae membrane (EIHM) compartment) (Zhang and Xu, 2014). The ER chaperone LHS1 is important for protein translocation across ER membrane as well as extracellular activities. Mutant *lhs1 M. oryzae* strain exhibit severely impaired conidiation, penetration and biotrophic invasion in susceptible rice (Yi et al., 2009). Consequently, *LHS1* was chosen as one of our HIGS candidates.

Fungal *Cytochrome P450 lanosterol C-14a- demethylase* (*CYP51*) genes are essential for ergosterol biosynthesis. Silencing of these genes can restrict fungal growth. CYP51 is also a target for systemic fungicides acting as demethylation inhibitors (DMIs). Fungicides like tebuconazole and triadimefon can bind CYP51 and interfere with fungal membrane integrity (Yoshida, 1993; Deng et al., 2007; Koch et al., 2013). It has been shown that silencing *CYP51* in *Arabidopsis* and barley confers strong resistance to *Fusarium* species (Koch et al., 2013). As *M. oryzae* contains two homologous *CYP51* genes (designated as *CYP51A* and *CYP51B*), they were both targeted simultaneously for our HIGS assay.

**FIGURE 1:**
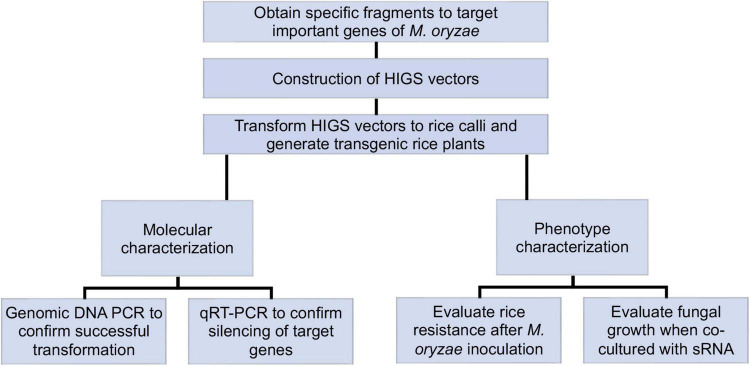
Schematic overview of host-induced gene silencing (HIGS) experimental workflow. Specific fragments were chosen and inserted into HIGS vectors to target important genes of *Magnaporthe oryzae*. Transgenic rice was generated by transforming HIGS vectors into rice calli. Transgenic rice was then subjected to molecular and phenotype characterization. Positive transgenic rice was first confirmed through genotyping. Then transgenic rice was inoculated with *M. oryzae* for fungal resistance evaluation. Relative transcription of fungal target genes from infected transgenic rice leaves was analyzed through qRT-PCR. Furthermore, *M. oryzae* was co-cultured with sRNA derived from target genes for fungal growth assay.

Figure 1 describes the experimental workflow that was applied to this research. Certain fragments of target fungal genes were cloned and inserted into HIGS destination vectors individually. Five HIGS vectors (one with both *CYP51A* and *CYP51B* fragments*)* were then transformed to rice calli respectively to generate transgenic rice. Molecular and phenotype characterization of HIGS plants were conducted to confirm positive transgenic rice and evaluate resistance to *M. oryzae*. *M. oryzae* was further co-cultured with small RNA to investigate the potential mechanism that underlies HIGS (Figure 1).

### Five host-induced gene silencing vectors were constructed targeting six fungal genes respectively

Target fragments from six candidate fungal genes were selected based on the criteria of presence of certain sequence motifs, optimal GC content, differential thermal stability, absence of poly-asparagine (polyN) sequence and low complexity. Specificity of those fragments was confirmed through a sequence similarity search on the rice genome annotation database^8^, and no homologous sequences were found. Information on candidate genes and target fragments is shown in Table 1.

PCR products were obtained for six target fragments by using cDNA of *M. oryzae* strain 70-15 as template. Fragments of *CYP51A* and *CYP51B* were linked together through joining PCR (Supplementary Figure 1). In total, five fragments were prepared for HIGS vector construction (Table 1). Using the “Gateway cloning system,” all were first cloned into the entry vector pDONR221 and then moved into the HIGS destination vector individually (Zhao et al., 2021). After recombination cloning, each target fragment was inserted into two regions of the destination vector flanked by recombination sites. Those insertions were designed to be in opposite directions, enabling the formation of inverted repeats (Miki and Shimamoto, 2004). In total, five different HIGS vectors targeting 6 fungal genes were constructed for rice transformation.

### Generation of transgenic rice plants expressing host-induced gene silencing constructs

**FIGURE 2:**
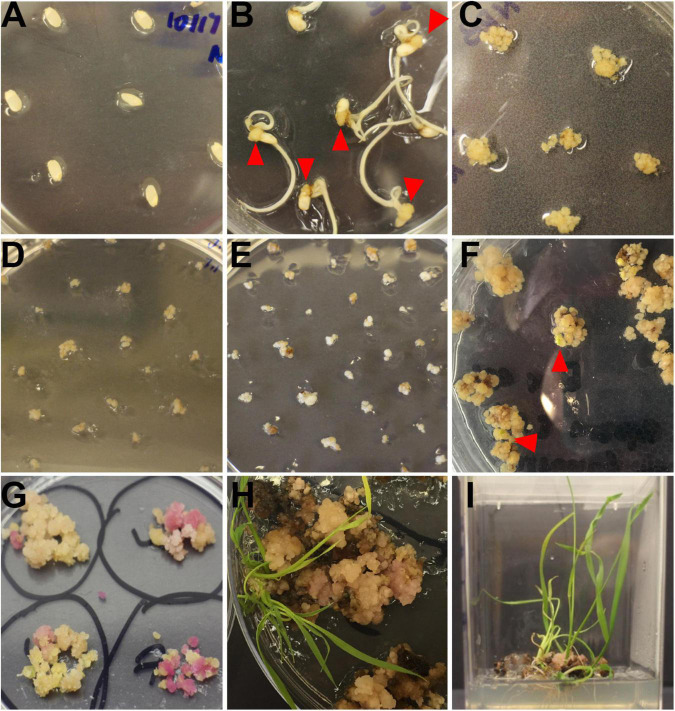
Workflow of rice transformation. **(A)** Seeds of Nipponbare on callus induction media. **(B)** Induced rice calli (red triangle) on callus induction media. **(C)** Rice calli used for transformation on fresh callus induction media. **(D)** Transformed calli co-cultured with *Agrobacteria*, calli were covered by *Agrobacteria*. **(E)** Rice calli on hygromycin selection media. Non-transformed calli turned dark brown and died while transformed calli grew fresh white colored calli. **(F)** Rice calli on shoot regeneration media. Some calli were ready to regenerate rice shoots (red triangle). **(G)** Rice calli on shoot regeneration media. Positively transformed calli turned red and were ready to generate shoots. **(H)** Regenerated rice shoots from transformed calli on shoot reaeration media. **(I)** Rice calli with regenerated shoots and roots on root regeneration media.

**FIGURE 3:**
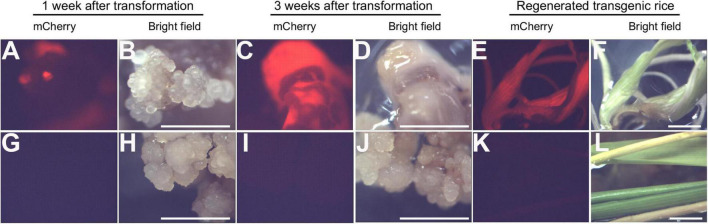
Positive transgenic rice confirmed through epifluorescence microscopy. **(A–F)** Transgenic rice calli **(A–D)** and seedling **(E,F)** after 1 week of transformation **(A,B)**, 3 weeks of transformation **(C,D)**, and shoot regeneration **(E,F)**. **(G–L)**. Non-transformed control rice calli **(G–J)** and seedling **(K,L)** after 1 week of transformation **(G,H)**, 3 weeks of transformation **(I,J)**, and shoot regeneration **(K,L)**. Scale bar = 1 cm.

**FIGURE 4:**
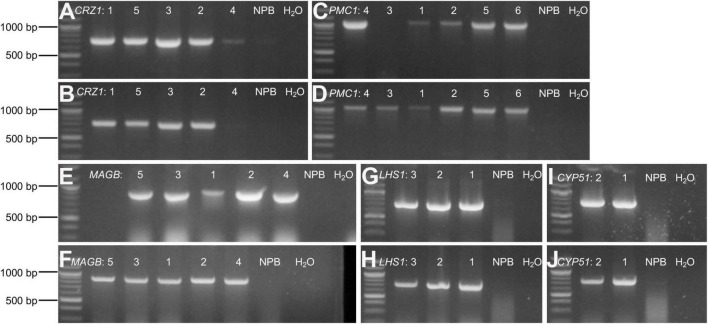
Confirm positive T0 transgenic rice through gDNA PCR. **(A–J)** Agarose gel electrophoresis for amplified target fragments of candidate HIGS gene *CRZ1* **(A,B)**, *PMC1* **(C,D)**, *MAGB* **(E,F)**, *LHS1* **(G,H)**, and *CYP51* **(I,J)**. Genomic DNA isolated from T0 transgenic rice leaves was used as templates. Primer pairs of “gus3 + corresponding target gene reverse primer” **(A,C,E,G,I)** and “gus4 + corresponding target gene reverse primer” **(B, D, F, H, J)** were used to amplify target fragments on both sides of *Gus* linker. Quick-Load Purple 1 kb Plus DNA Ladder from NEB was used as the DNA ladder. Arabic numbers were assigned to different rice plant lines representing individual transgenic event. Wild type Nipponbare rice plants as well as distilled water were used as negative control. The same assays were conducted to screen positive transgenic T1 and T2 generation rice plants.

Rice calli were induced from Nipponbare seeds (Figures 2A–C). Following *Agrobacterium* mediated transformation (Figures 2D,E), transgenic rice seedlings were generated (Figure 2F,G, H,I). mCherry fluorescence signal was visibly detected in positive transgenic calli as well as in regenerated rice shoots and roots (Figure 3). T0 generation transgenic rice were grown in the green house, and the presence of the inserted target fragments was confirmed by genomic DNA PCR. Inverted repeat sequences on both sides of *Gus* linker were amplified from transgenic T0 plants using different primer sets (Figure 4). All transgenic rice plants showed normal growth pattern compared to wild type Nipponbare.

### Expression of specific host-induced gene silencing vectors in transgenic rice confers resistance to *Magnaporthe oryzae*

Following self-pollination, T1 and T2 generation rice plants were obtained for pathogenicity assays. All transgenic rice derived from different HIGS constructs appeared healthy and demonstrated normal growth phenotype. Positive transgenic rice plants were confirmed through mCherry fluorescence detection as well as genomic DNA PCR. Negative plants segregating from each line without carrying the corresponding HIGS fragments were used as negative controls, as were wild type Nipponbare plants. Fully expanded rice leaves were infected with *M. oryzae* strain KJ-201 under favorable conditions, and necrotic lesion size analyzed a week later.

**FIGURE 5:**
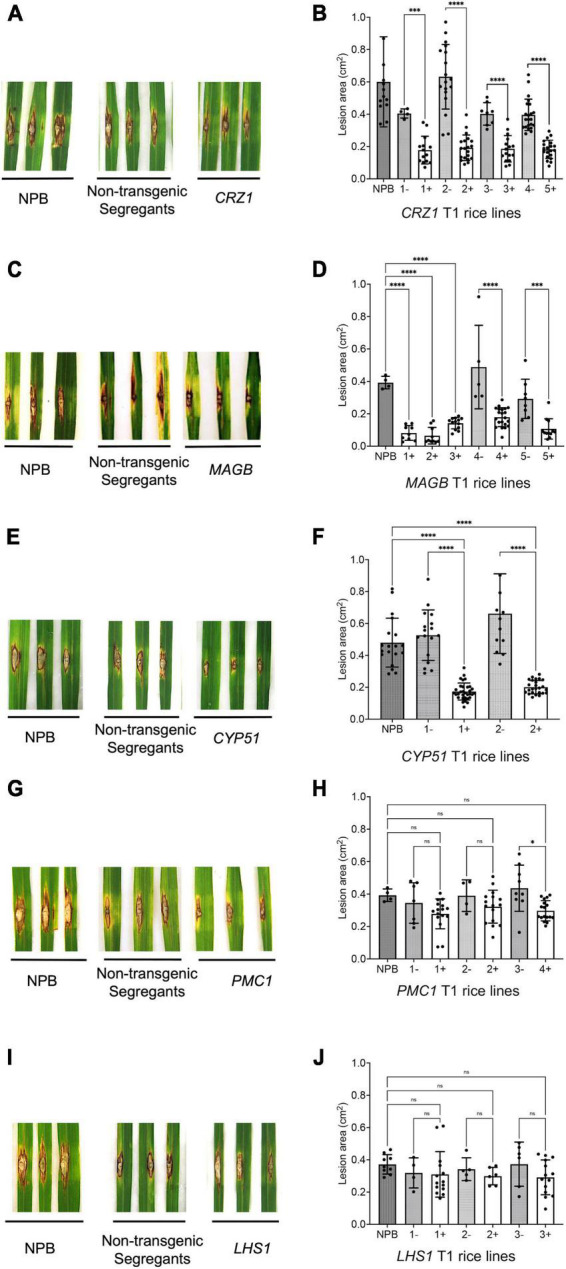
Certain host-induced gene silencing (HIGS) transgenic rice (T1 generation) showed resistance to *Magnaporthe oryzae*. **(A–J)** Punch inoculation of HIGS transgenic rice plants targeting *M. oryzae* gene *CRZ1* **(A,B)**, *MAGB* **(C,D)**, *CYP51* **(E,F)**, *PMC1* **(G,H)** and *LHS1* **(I,J)**. Wild type Nipponbare (NPB) and non-transgenic segregants derived from corresponding T0 plants were used as negative control. Lesion area was measured and analyzed statistically **(B,D,F,H,J)** using multiple rice lines. The symbol “+” represents positive transgenic plants while “-” represents non-transgenic segregants. Ordinary one-way ANOVA and Turkey’s multiple comparison were used for statistics analysis. *P < 0.0332, ^***^P < 0.0002, ^****^P < 0.0001, ns: not significant.

For T1 generation rice derived from HIGS vectors aiming to silence the fungal genes *CRZ1, MAGB* and *CYP51*, the lesion size on positive transgenic plants were significantly smaller compared to non-transgenic (negative) segregants plants and the wild type Nipponbare control (Figure 5). Five different lines were tested for HIGS plants derived from *CRZ1* gene (lines 1, 2, 3, 4, 5), five lines for *MAGB* gene (lines 1, 2, 3, 4, and 5) and two lines for *CYP51* gene (lines 1 and 2). For *CRZ1*, the lesion area was reduced 56.0%, 69.6%, 53.7% and 53.9%, respectively, for intra-line comparisons of lines “1”, “2”, “3” and crossline comparison between “4 and 5”. The lesions were 78.5% smaller for positive *MAGB* lines “1”, “2”, “3” compared with wild type and 65.0% and 63.5% smaller for intra-line (positive vs negative) comparison for line “4” and “5”, respectively. In addition, the *CYP51* lines “1” and “2” also showed a 67.1% and 69.7% reduction of lesion area compared with the non-transgenic segregants. However, for transgenic rice derived from HIGS vectors aiming to silence *PMC1* and *LHS1*, no significant differences for lesion size among positive transgenic rice, non-transgenic segregants and wild type Nipponbare were observed (Figure 5).

**FIGURE 6:**
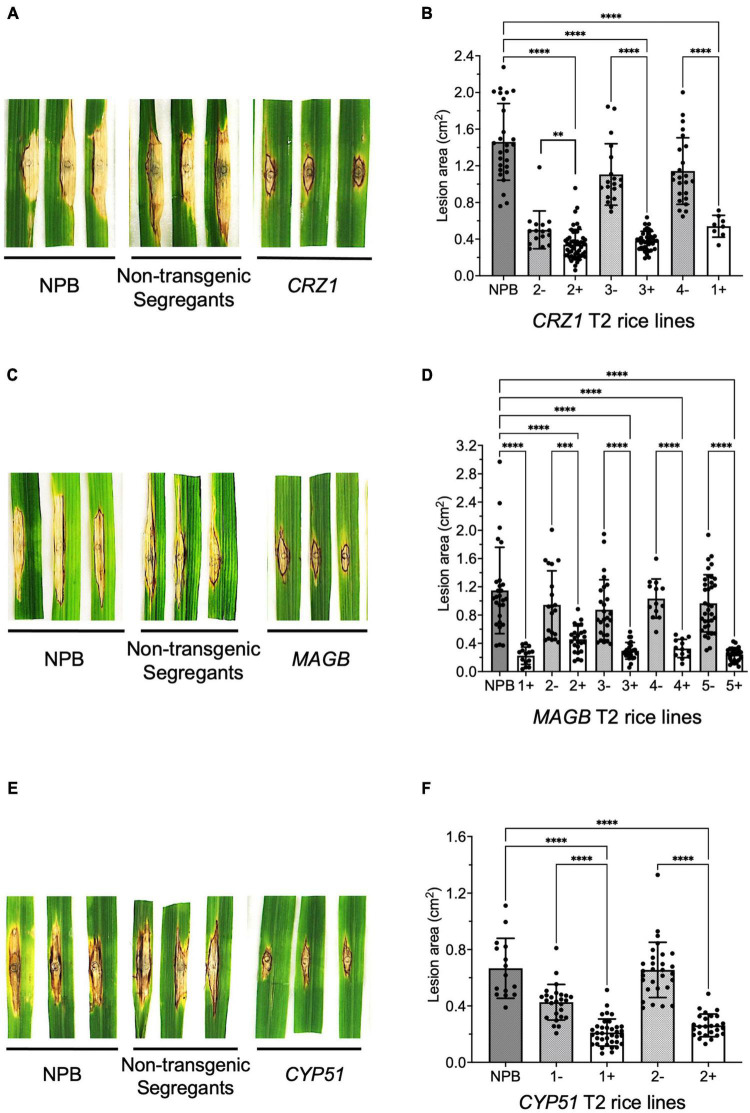
Confirm resistant host-induced gene silencing (HIGS) transgenic rice in T2 generation. **(A–F)** Punch inoculation of HIGS transgenic rice plants targeting *Magnaporthe oryzae* gene *CRZ1* **(A,B)**, *MAGB* **(C,D)**, *CYP51* **(E,F)**. Wild type Nipponbare (NPB) and non-transgenic segregants derived from corresponding T1 plants were used as negative control. Lesion area was measured and analyzed statistically **(B,D,F)** using multiple rice lines. The symbol “+” represents positive transgenic plants while “-” represents non-transgenic segregants. Ordinary one-way ANOVA and Turkey’s multiple comparison were used for statistics analysis. ^***^P < 0.0002, ^****^P < 0.0001.

To confirm resistance phenotype obtained from the T1 generation, T2 rice plants targeting *CRZ1, MAGB* and *CYP51* were grown and inoculated with *M. oryzae* again. Similar results were obtained (Figure 6). For *CRZ1* transgenic plants, the lesion area of line “1” were 52.7% smaller than the negative line “4” while the intra-line reductions were 32.2% for line “2” and 65.4% for line “3”. For *MAGB* transgenic plants, the lesion area was reduced 80.5% when comparing line “1” with wild type. The reduction of lesion areas was 51.5%, 66.4%, 68.5% and 75.1% when compared positive transgenic rice line “2”, “3”, “4”, and “5” with their non-transgenic segregants respectively. For *CYP51* transgenic rice, the lesions were 50.5% and 60.0% smaller for line “1” and “2” when compared with corresponding non-transgenic segregants (Figure 6).

### Target fungal genes are repressed in *Magnaporthe oryzae* infected host-induced gene silencing-expressing transgenic rice plants

**FIGURE 7:**
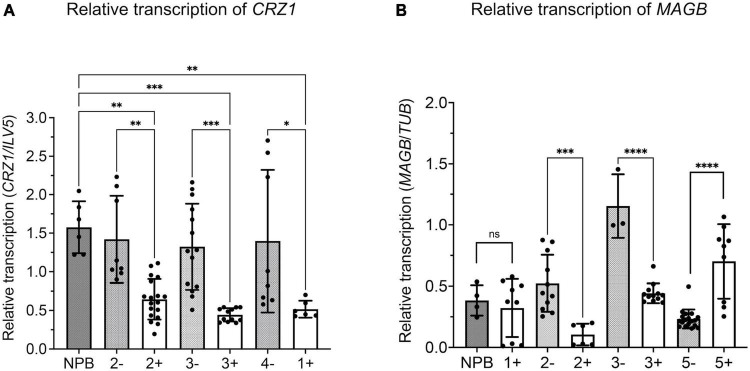
Relative transcription of target gene reduced in *Magnaporthe oryzae* invading host-induced gene silencing (HIGS) transgenic rice targeting *CRZ1* and *MAGB*. A,B Analysis of relative transcription of fungal gene *CRZ1* **(A)** and *MAGB* **(B)** through quantitative RT-PCR. Fungal gene *ILV5* **(A)** or β-*Tubulin* **(B)** were used as the reference gene. Multiple rice lines inoculated with *M. oryzae* were tested. “+” represents positive transgenic rice while “-” represents non-transgenic segregants. cDNA was generated from total RNA isolated from *M. oryzae*-inoculated rice leaves after 14 days. Bars represent mean values ± SDs of at least three independent sample collections. (*P < 0.0332, ^**^P < 0.0021, ^***^P < 0.0002, ^****^P < 0.0001; Tukey test).

Quantitative real time-PCR analysis was carried out to verify silencing of target fungal genes. *M. oryzae* was inoculated on rice leaves and fungal transcripts were analyzed 14 days after inoculation (Figure 7). Results showed reduced levels of *CRZ1* and *MAGB* endogenous transcripts in positive transgenic rice compared to non-transgenic segregants as well as wild type Nipponbare. For gene *CRZ1*, the transcription level was reduced by 63.1, 54.6, and 66.5% when positive transgenic line “1”, “2”, and 3” were compared with non-transgenic segregants (Figure 7A). For gene *MAGB*, the transcription was reduced 89.1% in line “2” and 67.0% in line “3” using *ILV5* as the internal reference, while the reductions were 79.9 and 61.7%, respectively, when β-*Tubulin* was used as fungal reference. However, reduced *MAGB* transcription was not detected for line 5 (Supplementary Figure 2A and Figure 7B). In addition, no significant difference was observed for *CYP51* transcription between transgenic rice plants and wildtype control (Supplementary figure 2B). Overall, for HIGS plants targeting *CRZ1* and *MAGB*, the qRT-PCR results are consistent with the hypothesis that impairment of the pathogen’s ability to cause disease on rice leaves is related to reduced fungal gene expression induced by HIGS.

### Incubation with small RNA derived from fungal genes inhibits growth of *Magnaporthe oryzae*

**FIGURE 8:**
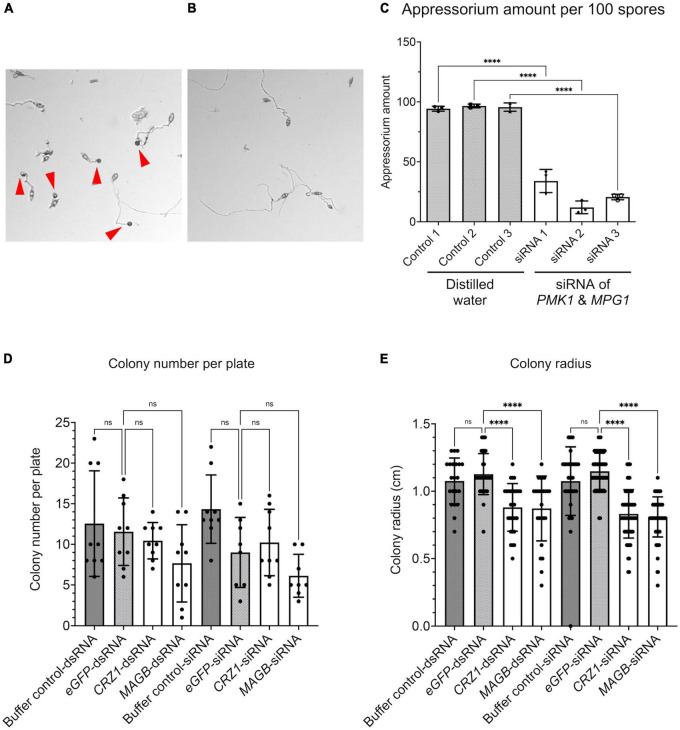
RNA silencing signals can be absorbed by *Magnaporthe oryzae* resulting in functional collapse. **(A)** Spore suspension treated with distilled water. Appressoria (red arrows point to appressoria) were formed at tips of germ tube. **(B)** Spore suspension treated with siRNA derived from *PMK1&MPG1*, less appressoria were formed. **(C)** Appressorium amount after water treatment and *PMK1&MPG1* siRNA treatment. Ordinary one-way ANOVA and Turkey’s multiple comparison were used for statistics analysis. **(D)** No statistical difference was observed considering fungal colony number when spore suspensions were treated with buffer control, dsRNA/siRNA derived from *eGFP, CRZ1* or *MAGB*. **(E)** Treatment of dsRNA/siRNA derived from *CRZ1/MAGB* reduced fungal colony radius when compared to spore suspensions treated with buffer control or *eGFP*-dsRNA/siRNA. Buffer solution was negative control solution that went through the whole process of dsRNA/siRNA synthesis but without templates. **** *P* < 0.0001, ns: not significant.

We show HIGS can confer rice resistance to *M. oryzae*, however, how are small RNA transferred from the plant cell to fungal cell and trigger silencing of target genes is not fully understood. To investigate whether fungal cells can take up dsRNA and siRNA targeting the fungal genes, dsRNA derived from fragments of *PMK1*, *MPG1* as well as *CYP51*, *CRZ1*, and *MAGB* were synthesized *in vitro* using the T7 RNA polymerase transcription system (Supplementary Figure 3). Furthermore, siRNA targeting each gene were processed using short cut RNase III (Supplementary Figure 3). Fungal spore suspensions were incubated with synthesized dsRNA or siRNA and then subjected to morphological evaluation. *PMK1* and *MPG1* are genes important for appressorium formation in *M. oryzae. PMK1* is a mitogen-activated protein (MAP) kinase gene regulating formation of appressorium and infectious hyphal growth of *M. oryzae* (Bruno et al., 2004). *MPG1* is a hydrophobin-encoding gene. Loss of function mutant shows reduced virulence, conidiation and appressorium formation (Soanes et al., 2002). siRNA derived from *M. oryzae PMK1* and *MPG1* dramatically interfered with appressorium formation and reduced formation by around 70% (Figures 8A–C). Total number of fungal colonies on dsRNA and siRNA treated plates were not significantly different from control plates (Figure 8D and Supplementary Figure 4). Fungi treated with buffer solution as well as dsRNA/siRNA derived from *eGFP* were used as controls. dsRNA as well as siRNA derived from *CRZ1, MAGB and CYP51* greatly reduced fungal colony radius (Figure 8E and Supplementary Figure 4), indicating that both naked dsRNA and siRNA may move into fungal cells and induce RNAi, resulting in growth inhibition.

## Discussion

As a novel RNAi based technology for efficient control of fungal pathogens and other pests, our results show that HIGS can be effective at significantly reducing rice blast disease caused by *M. oryzae*. In general, we were able to achieve greater than 50% reductions in symptoms and in some cases 80% reduction in lesion size when certain genes were targeted. *M. oryzae* as a hemibiotroph pathogen initially absorbs nutrients from living cells and then kills host cells in order to live necrotrophically on the dead tissues. We do not know which infection stage is most effective for HIGS, but during both stages, RNA silencing signals likely have the opportunity to move from plant cells to invading fungal cells and induce silencing of target genes. Choosing the right fungal gene to silence and gene segment is vital. Genes sharing duplicate function with others, performing minor effects for fungal growth and infection or genes capable of rapid evolution of substitution such as effectors are not likely to be appropriate HIGS candidates. Previous HIGS studies (Nowara et al., 2010; Yin et al., 2011; Zhang et al., 2012; Panwar et al., 2013; Hu et al., 2015; Zhou et al., 2016) have targeted many key fungal genes playing important roles in growth and development such as genes encoding calcineurin, MAP kinases, F-box proteins, chitin synthases, RNA polymerases, ubiquitin E3 ligases and others all of which represented good HIGS candidates. HIGS technology has been evaluated for rice blast disease. Zhu et al. used Brome mosaic virus (BMV) mediated HIGS to show that fungal pathogenicity genes: *MoABC1*, *MoMAC1* and *MoPMK1* are good targets for HIGS conferred disease resistance. However, the assay was analyzed through a transient expression system (Zhu et al., 2017). Targeting bZIP transcription factor encoding gene *MoAP1* as well as the actin cross-linking protein Fimbrin encoding gene *MoFim1* also reduced rice blast disease (Guo et al., 2019; Li et al., 2020). Here we tested 6 other target genes and found that *CRZ1*, *MAGB* and *CYP51* genes important for signal transduction and membrane integrity, are also viable and effective targets for HIGS. In the future, multiple fungal genes could be targeted together through co-transformation or crossing rice lines to provide rice with more durable and robust resistance to *M. oryzae*.

It is also important to identify a suitable fragment of the candidate gene as the target sequence because different gene fragments may perform with different efficiency to generate siRNA leading to RNAi (Baldwin et al., 2018). In this study, transgenic rice derived from HIGS vectors targeting *LHS1* and *PMC1* did not exhibit resistance to *M. oryzae*. One reason may be that proper gene function is only affected when the gene expression is totally eliminated. However, it may be also due to inappropriate selection of target fragments for those two genes such that no or very little silencing was accomplished to generate an observable phenotype. Another aspect of note was for HIGS plants targeting fungal gene *CYP51.* While disease resistance phenotype was observed in both T1 and T2 generation (Figures 5, 6), the relative transcription of *CYP51* in infected HIGS plants was not altered (Supplementary Figure 2B). This may have resulted from several issues such as DNA contamination during RNA extraction, interference in the qPCR process or small RNA off targeting in the HIGS system. However, the most likely reason may be poor design of *CYP51* primers used in RT-qPCR analysis. *CYP51* is one of the most ancient and conserved P450s across kingdoms (Yoshida et al., 1997; Debeljak et al., 2003; Kim et al., 2005; Becher and Wirsel, 2012). Rice also contains multiple *CYP51* genes (Nelson et al., 2004; Lepesheva and Waterman, 2007). Although we carefully chose the fungal specific *CYP51* fragment to construct the HIGS vector, the primers used in qPCR analysis may not have been specific enough to amplify the fungal *CYP51* fragment exclusively. Rice *CYP51* may also have been amplified causing the unexpected qPCR results. Every consideration needs to be taken when choosing genes conserved among plants and fungi as HIGS targets. On the other hand, targeting a conserved fragment across different pathogenic fungi may confer HIGS plants with broad spectrum resistance to pathogens.

Although several studies have demonstrated the possibility of using HIGS as a novel strategy for disease control, few have provided insight into the underlying signal mechanism(s). What is the silencing signal (dsRNA or siRNA)? How do small RNA signals get sorted and transported between plant to pathogen without degradation? As demonstrated by this study, siRNA and dsRNA can be absorbed by fungal mycelium and may induce silencing of target genes. Similar results have also been obtained for *Aspergillus nidulans* (Kalleda et al., 2013) as well as *Rhizoctonia solani* (Zhou et al., 2016). For *M. oryzae*, silencing of *MoAP1* can be achieved through *in vitro* feeding of artificial small RNA targeting *MoAP1.* This resulted in impaired fungal development (Guo et al., 2019). Those interesting results further point out the potential for using small RNAs as fungicide for disease control.

Recently, Hailing Jin’s group has demonstrated that extracellular vesicles carrying siRNA can move between fungal and plant cells (Jin, 2008; Weiberg et al., 2013; Wang et al., 2016; Cai et al., 2018). Nevertheless, the evidence is still limited, and it is not clear how such vesicles are directed to cross into invading pathogen cells and maintain optimal concentrations to induce RNAi. Important proteins involved in silencing signal sorting, transportation, recognition, absorption and protection from degradation are worthy of further research. Using fluorescence or radioactively labeled siRNA may help to decipher the entire process of HIGS. Inhibitors such as secramine that block plant vesicle secretion may further confirm the role of vesicles for small RNA communication (Pelish et al., 2006; von Kleist and Haucke, 2012). For practical application, artificial vesicles containing siRNA as well as synthesized naked siRNA (Guo et al., 2019) may be suitable as bio-fungicides to control plant diseases. For example, spraying dsRNA targeting *CYP51* in *Fusarium graminearum* provided some disease control on barley (Koch et al., 2016). Spray induced gene silencing (SIGS) has also been demonstrated for rice-*M. oryzae* interaction system. Spray of dsRNA targeting fungal *MoDES1* induced silencing of *MoDES1* and conferred significant resistance against blast disease (Sarkar and Roy-Barman, 2021). Above all, our study demonstrates the effectiveness of using HIGS in rice blast disease control and highlights that targets and silencing fragments need to be carefully evaluated. Moreover, to facilitate effective application, further studies are needed to address the underlying mechanism(s) for cross-kingdom siRNA communication.

## Data availability statement

The original contributions presented in this study are included in the article/Supplementary material, further inquiries can be directed to the corresponding author.

## Author contributions

MW and RD designed the experiments. MW carried out experiments and analyzed data. MW and RD wrote the manuscript. Both authors contributed to the article and approved the submitted version.

## Footnotes

1
fungi.ensembl.org/index.html

2
rnaidesigner.thermofisher.com/rnaiexpress/design.do

3
rice.uga.edu/analyses_search_blast.shtml

4
bsw3.naist.jp/simamoto/pANDA/real/gus_linker.htm

5
https://www.megasoftware.net/mega4/mega.html

6
rsbweb.nih.gov/ij/

7
www.graphpad.com/

8
rice.uga.edu/analyses_search_blast.shtml
