# Supplementary material for: Host induced gene silencing of Magnaporthe oryzae by targeting pathogenicity and development genes to control rice blast disease
Source: Front Plant Sci. 2022 Aug 11;13:959641. doi: 10.3389/fpls.2022.959641 (PMC9403838; doi:10.3389/fpls.2022.959641)
Supplement: Supplementary file 1 [file Data_Sheet_1.docx]

Supplementary Material

# Supplementary Table 1 Primers used in this study

| Primer Name | Function | Primer sequence |
| --- | --- | --- |
| Mg-*LHS1*-attb1-F | Vector Construction | GGGGACAAGTTTGTACAAAAAAGCAGGCTGGTGGTGACGCTTTGAACTA |
| Mg-*LHS1*-attb2-R | Vector Construction | GGGGACCACTTTGTACAAGAAAGCTGGGTCTGAAGCCAGCACCAAATAC |
| Mg-CRZ*1*-attb1-F | Vector Construction | GGGGACAAGTTTGTACAAAAAAGCAGGCTATAAGAGGCGTCAGTCCACC |
| Mg-CRZ*1*-attb2-R | Vector Construction | GGGGACCACTTTGTACAAGAAAGCTGGGTCCAGATTCTTTGCCGTTCCA |
| Mg-*PMC1*-attb1-F | Vector Construction | GGGGACAAGTTTGTACAAAAAAGCAGGCTCCAGACAAGCTCGCCTATCTCGCCG |
| Mg-*PMC1*-attb2-R | Vector Construction | GGGGACCACTTTGTACAAGAAAGCTGGGTCGTCCTCTCCGGTAGCCTGTTGTCC |
| *MAGB*-attb1-F | Vector Construction | GGGGACAAGTTTGTACAAAAAAGCAGGCTAGCTCAAGAGGGACAGGTTG |
| *MAG*B-attb2-R | Vector Construction | GGGGACCACTTTGTACAAGAAAGCTGGGTGCTGATCGTACTCGGAAATG |
| Mg-*CYP51A*-F | Vector Construction | GAGTCCTCACCCGTCTTCCAAGCCGGCA |
| Mg-*CYP51A*-R | Vector Construction | TGTTGCCGTATCTTCTTGATCGTCCTTCTG |
| Mg-*CYP51B*-F | Vector Construction | ATCAAGAAGATACGGCAACATCTTTACCTT |
| Mg-*CYP51B*-R | Vector Construction | TTCATAAACTTCTTTTGCTCCATCAGCT |
| Mg-*CYP51A*-attb1-F | Vector Construction | GGGGACAAGTTTGTACAAAAAAGCAGGCTGAGTCCTCACCCGTCTTCCA |
| Mg-*CYP51B*-attb2-R | Vector Construction | GGGGACCACTTTGTACAAGAAAGCTGGGTTTCATAAACTTCTTTTGCTCCAT |
| *GUS 3* | gDNA PCR | CAGTCCATTAATGCGTGGTCGT |
| *GUS 4* | gDNA PCR | TGTATCACCGCGTCTTTGATCG |
| T7-*PMK1*-F | sRNA Synthesis | TAATACGACTCACTATAGGG TACCTACACCACAGCTTT |
| T7-*PMK1*-R | sRNA Synthesis | TAATACGACTCACTATAGGGTTACCGCATAATTTCCTG |
| T7-*MPG1*-F | sRNA Synthesis | TAATACGACTCACTATAGGG ATGTTCTCCCTCAAGACC |
| T7-*MPG1*-R | sRNA Synthesis | TAATACGACTCACTATAGGG CAAGCACAAG TCACGAGC |
| T7-*eGFP*-F | sRNA Synthesis | TAATACGACTCACTATAGGGATGGTGAGCAAGGGCG |
| T7-*eGFP*-R | sRNA Synthesis | TAATACGACTCACTATAGGGTGTACAGCTCGTCCATG |
| T7-*CYP51*-F | sRNA Synthesis | TAATACGACTCACTATAGGGCCAGACAAGCTCGCCTATCTCGCCG |
| T7-*CYP51*-R | sRNA Synthesis | TAATACGACTCACTATAGGGCGTCCTCTCCGGTAGCCTGTTGTCC |
| T7-*CRZ1*-F | sRNA Synthesis | TAATACGACTCACTATAGGGATAAGAGGCGTCAGTCCACC |
| T7-*CRZ1*-R | sRNA Synthesis | TAATACGACTCACTATAGGGCCAGATTCTTTGCCGTTCCA |
| T7-*MAGB*-F | sRNA Synthesis | TAATACGACTCACTATAGGGAGCTCAAGAGGGACAGGTTG |
| T7-*MAGB*-R | sRNA Synthesis | TAATACGACTCACTATAGGGGCTGATCGTACTCGGAAATG |
| qPCR-*b-tub*-F | qPCR | CGCGGCCTCAAGATGTCGT |
| qPCR-*b-tub*-R | qPCR | GCCTCCTCCTCGTACTCCTCTTCC |
| qPCR-*CYP51A*-F | qPCR | CTCGAATGCGCGATGTTTAC |
| qPCR-*CYP51A*-R | qPCR | GTCTGGCTCTGCTTCTTCTT |
| qPCR-*CYP51B*-F | qPCR | GCCTTCGTCAGCTTCATCTT |
| qPCR-*CYP51B*-R | qPCR | CGTACTTCTTGGCCATGTCC |
| q-PCR-*MAGB*-F | qPCR | TCATGGCGGCTGTTAATGA |
| q-PCR-*MAGB*-R | qPCR | CTGAGAAATGAGGGTGGTAGTG |
| qPCR-*CRZ1*-F | qPCR | TTTCCTCAACTGCCACAGCAT |
| qPCR-*CRZ1*-R | qPCR | TACGCGTCCTCTATCCGGCG |
| qPCR-*ILV5*-F | qPCR | CCAGCTCTACGACTCGGTCAA |
| qPCR-*ILV5*-R | qPCR | AGTCGGGCTGGCTGTTGTAGT |

# Supplementary Figures


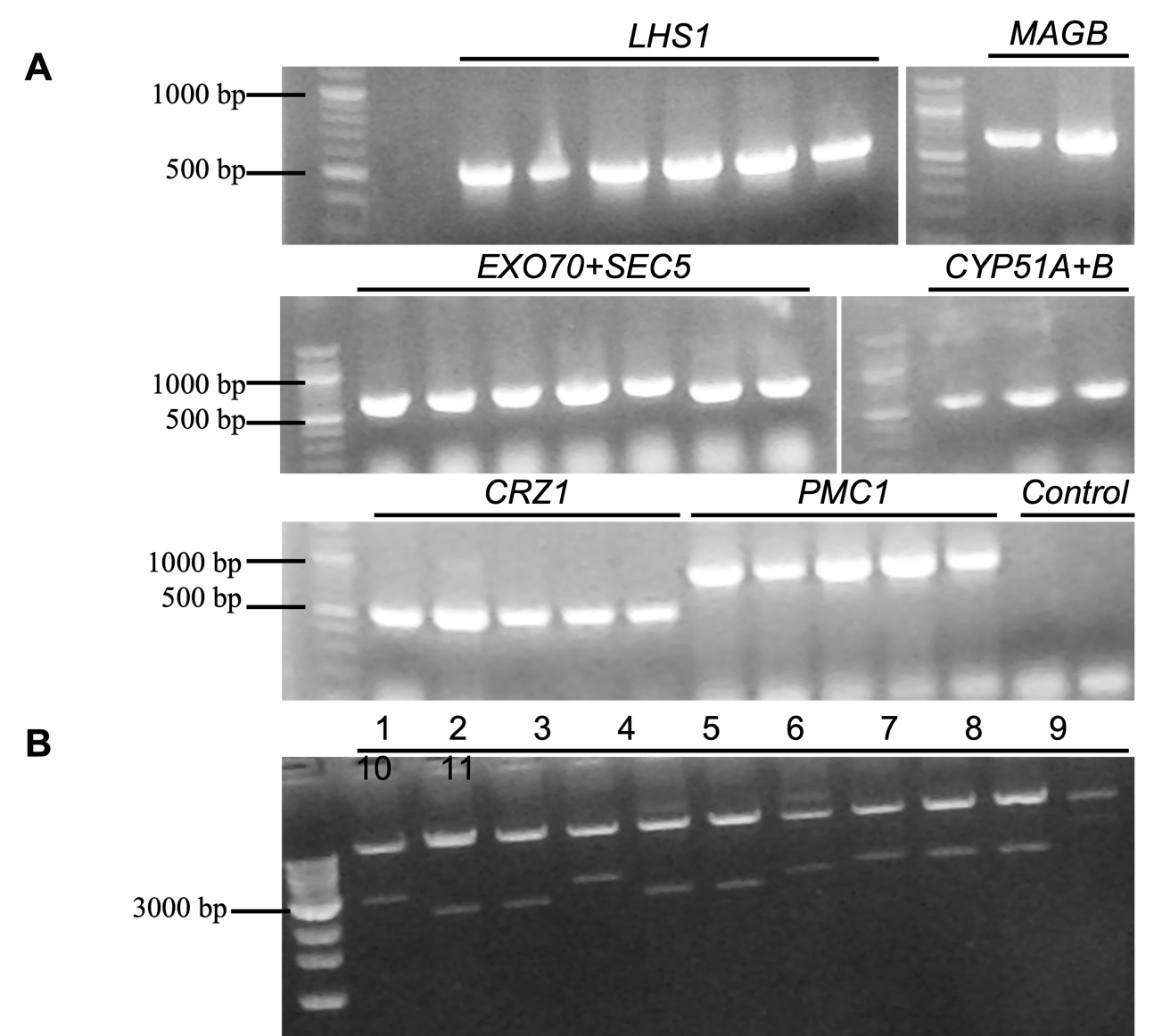


**Supplementary Figure 1.** Verification of the positive ENTRY constructs as well as HIGS constructs. (A) PCR products of target fragments using corresponding ENTRY constructs as templates. Distilled water was used as template for negative control. (B) Gel fraction of HIGS constructs after digestion with two restriction enzyme *Kpn* I & *Sac* I. (1, 4, 8, 9, 10: *PMC1*/pBDL03; 2, 3, 5, 6: *CRZ1*/pBDL03; 7: *MAGB*/pBDL03; 11: empty pBDL03 used as negative control)


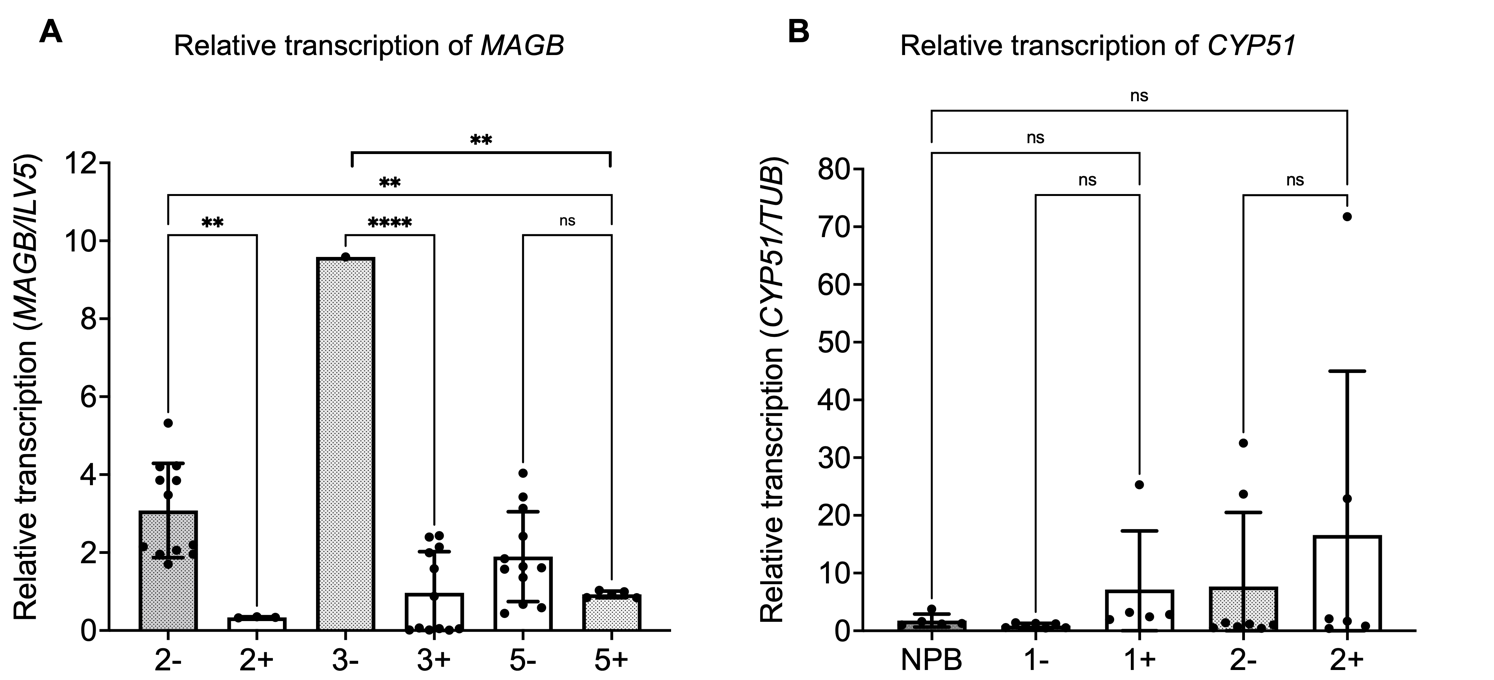


**Supplementary Figure 2.** Analysis of relative transcription of fungal *MAGB* and *CYP51* gene through quantitative RT-PCR using fungal gene *ILV5* or *Tub* as the reference gene. (A) Relative transcription of fungal *MAGB* using fungal gene *ILV5* as the reference gene. Three lines were tested. (B) Relative transcription of fungal *CYP51* using fungal gene *Tub* as the reference gene. Two lines were tested. “+” represents positive transgenic rice while “-” represents non-transgenic segregants. cDNA was generated from total RNA isolated from *M. oryzae*-inoculated rice leaves after 14 days. Bars represent mean values ± SDs of at least three independent sample collections. (**P<0.0021, ****P<0.0001; Tukey test)


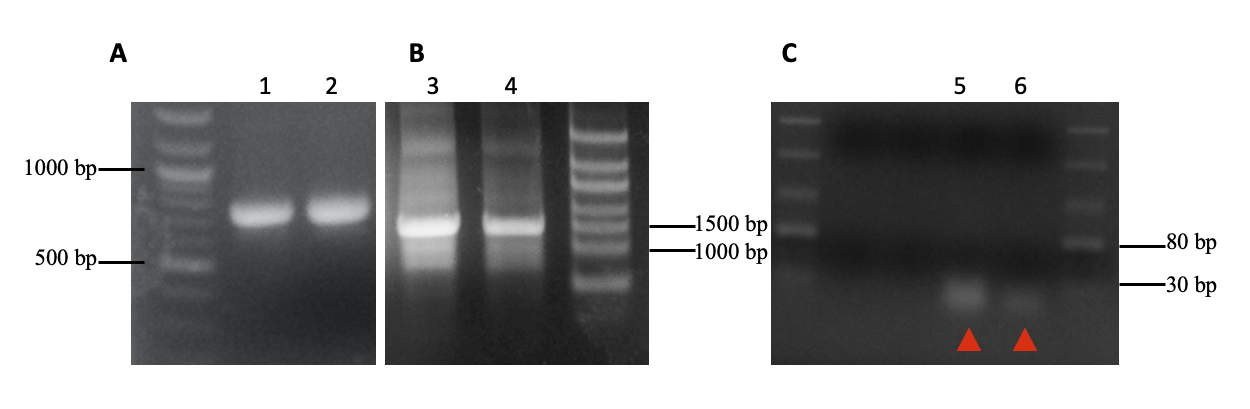


**Supplementary Figure 3.** Schematic process for dsRNA and siRNA synthesis. (A) 1-2: PCR products of control gene *eGFP*. (B) 3-4: Annealed dsRNA of *eGFP* through in vitro transcription. (C) 5-6: siRNA of *eGFP* after Rnase III digestion


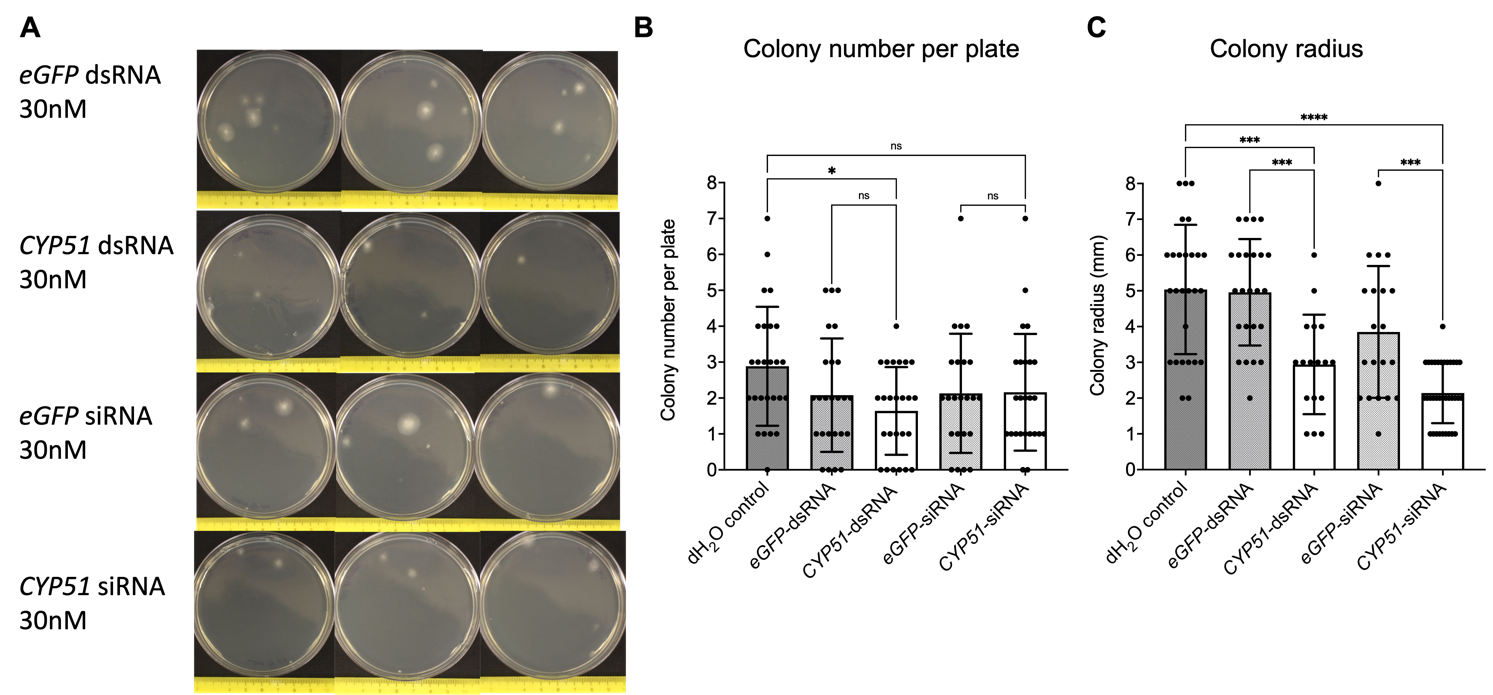


**Supplementary Figure 4.** RNA silencing signals may be absorbed by *M. oryzae*. (A) Colony phenotype treated with 30nM dsRNA/siRNA derived from *eGFP* as well as *CYP51*. (B) There is no significant difference in colony number on each plate when treated spore suspension with siRNA/dsRNA derived from *eGFP/CYP51*. (C) Colony radius of spores treated with siRNA/dsRNA derived from *CYP51* were smaller compared to spores treated with *eGFP*-siRNA/dsRNA (*P < 0.0332, ***P < 0.0002, ****P<0.0001; Tukey test).

**
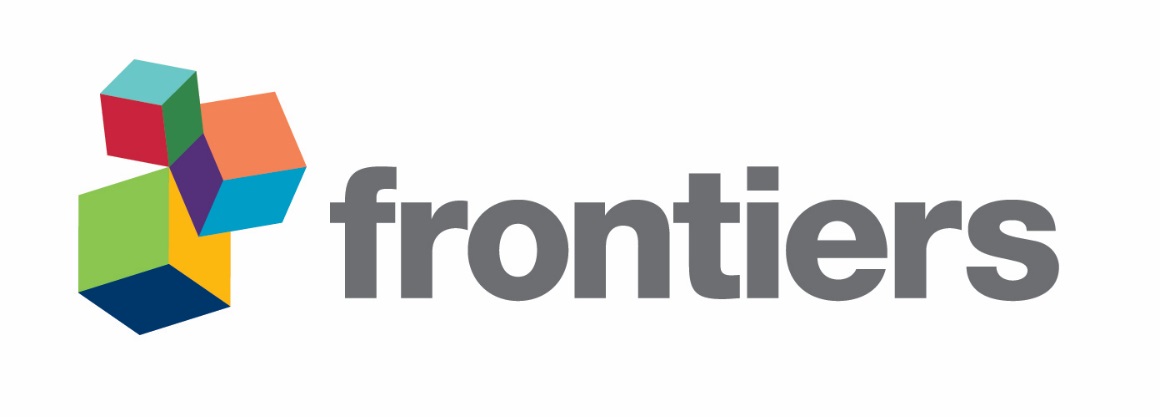
**
